# Novel Ocellatin Peptides Mitigate LPS-induced ROS Formation and NF-kB Activation in Microglia and Hippocampal Neurons

**DOI:** 10.1038/s41598-020-59665-1

**Published:** 2020-02-14

**Authors:** Nayara A. Sousa, Guilherme A. L. Oliveira, Ana Patrícia de Oliveira, André Luís F. Lopes, Bruno Iles, Kerolayne M. Nogueira, Thiago S. L. Araújo, Luan K. M. Souza, Alyne R. Araújo, Joilson Ramos-Jesus, Alexandra Plácido, Constança Amaral, Yuri D. M. Campelo, Eder Alves Barbosa, Camila C. Portugal, Renato Socodato, Andrea Lobo, Joao Relvas, Marcelo Bemquerer, Peter Eaton, José Roberto S. A. Leite, Jand Venes R. Medeiros

**Affiliations:** 1 Laboratório de Farmacologia da Inflamação e Doenças Gastrintestinais, Universidade Federal do Delta do Parnaíba, UFDPar, Piauí, Brazil; 2 Núcleo de Pesquisa em Biodiversidade e Biotecnologia, Universidade Federal do Piauí, UFPI, Piauí, Brazil; 3 Instituto de Educação Superior do Vale do Parnaíba, FAHESP/IESVAP/NRE, Parnaíba, Brazil; 4 LAQV/REQUIMTE, Departamento de Química e Bioquímica, Faculdade de Ciencias da Universidade do Porto, Porto, Portugal; 5 Instituto de Investigação e Inovação em Saúde and Instituto de Biologia Molecular e Celular (IBMC), Universidade do Porto, Porto, Portugal; 6 Instituto de Medicina Molecular, IMM, Universidade de Lisboa, Lisboa, Portugal; 7 Laboratório de Síntese e Análise de Biomoléculas, LSAB, Instituto de Química, UnB, Brasília, Brazil; 8 Embrapa Recursos Genéticos e Biotecnologia, Brasília, Brazil; 9 Núcleo de Pesquisa em Morfologia e Imunonologia Aplicada, NuPMIA, Área Morfologia, Faculdade de Medicina, UnB, Brasília, Brazil

**Keywords:** Peptides, Natural product synthesis, Pharmacodynamics

## Abstract

Cutaneous secretions of amphibians have bioactive compounds, such as peptides, with potential for biotechnological applications. Therefore, this study aimed to determine the primary structure and investigate peptides obtained from the cutaneous secretions of the amphibian, *Leptodactylus vastus*, as a source of bioactive molecules. The peptides obtained possessed the amino acid sequences, GVVDILKGAAKDLAGH and GVVDILKGAAKDLAGHLASKV, with monoisotopic masses of [M + H]^±^ = 1563.8 Da and [M + H]^±^ = 2062.4 Da, respectively. The molecules were characterized as peptides of the class of ocellatins and were named as Ocellatin-K1(1–16) and Ocellatin-K1(1–21). Functional analysis revealed that Ocellatin-K1(1–16) and Ocellatin-K1(1–21) showed weak antibacterial activity. However, treatment of mice with these ocellatins reduced the nitrite and malondialdehyde content. Moreover, superoxide dismutase enzymatic activity and glutathione concentration were increased in the hippocampus of mice. In addition, Ocellatin-K1(1–16) and Ocellatin-K1(1–21) were effective in impairing lipopolysaccharide (LPS)-induced reactive oxygen species (ROS) formation and NF-kB activation in living microglia. We incubated hippocampal neurons with microglial conditioned media treated with LPS and LPS in the presence of Ocellatin-K1(1–16) and Ocellatin-K1(1–21) and observed that both peptides reduced the oxidative stress in hippocampal neurons. Furthermore, these ocellatins demonstrated low cytotoxicity towards erythrocytes. These functional properties suggest possible to neuromodulatory therapeutic applications.

## Introduction

The skin of amphibians has been the subject of interest and study of several research groups as well as pharmaceutical industries, due to the abundance and diversity of bioactive molecules with potential biotechnological applications, especially for the production of new drugs^1^. The characteristic way of living of amphibians is divided between aquatic and the terrestrial environment^2^. They possess a highly sensitive skin that is essential to its respiration and is highly vulnerable to environmental aggressions, such as desiccation, attack of microorganisms, ultraviolet radiation, and injuries^3^. This vulnerability has culminated in the development of an innate defense system as a survival strategy based on the expression, production, accumulation, and secretion of bioactive molecules, such as peptides, by glands located in the dermis of these animals^4^. Nevertheless, this vulnerability may reappear in the presence of a global biotic threat such as the chytridiomycosis that promoted a huge reduction of Amphibian biodiversity affecting many species of the Leptodactylidae family^5^.

Amphibian’s peptides are attractive candidates for investigating biological activities that may reveal detailed molecular defence mechanisms and high levels of functional diversity. They are known to function as antihypertensives and vasodilators, opioids, peptidase inhibitors, neuropeptides, peptides for wound healing, nitric oxide inhibitors, insulin releasers, myotropics, antitumoral, antimicrobial, and antioxidant peptides^6^; all these properties affect potential predators and pathogens^3^. Although a number of such defense peptides have been reported against biological injuries, very few peptides against abiotic injuries, such as those caused by exposure to ultraviolet radiation have been studied.

In amphibians, exposure to ultraviolet radiation in the sensitive corneous area of the skin together with the difference in oxygen availability caused by the transition between the aquatic and terrestrial environment results in an accelerated endogenous production of reactive oxygen species (ROS)^7^. Under these conditions, when the environmental oxygen concentration is higher, the skin of the amphibians consumes more absorbed oxygen instead of satisfying the oxygen demands of other tissues. In addition, loss of body water is associated with increased oxidative damage^2^. Thus, one may suggest that the skin of amphibians can contribute to homeostasis against accelerated oxidative stress developed during changing environmental conditions for their survival. Among the strategies already known to protect amphibians from ultraviolet light are the proteins Melanopsin present in skin pigment cells^8^ and Pheomelanin found in the dorsal skin of *Hymenochirus boettgeri*^9^. Additionally, some studies have demonstrated antioxidant potential of peptides extracted from the cutaneous secretion of frogs, such as antioxidin-RL with a strong free radical scavenging ability^2^ and antioxidin-I that substantially attenuates the hypoxia-induced ROS production in living microglia, suggesting a potential neuroprotective role for this peptide^10^.

Oxidative stress is a cellular or physiological condition with a high concentration of ROS that causes molecular damage to cellular structures^11^. Although cells contain a number of antioxidant defenses for minimizing ROS fluctuations, situations where ROS generation often exceed the antioxidant capacity of cells are correlated with the onset and progression of many diseases through mutations of DNA, protein oxidation, and lipid peroxidation with consequent functional alterations and loss of vital functions in several tissues or organs^12,13^. In this context, considering that the neuroanatomic region of the brain is highly vulnerable to oxidative stress, molecules such as amphibian antioxidant peptides, which rapidly exert their biological functions by eliminating free radicals within several seconds may serve as promising neuroprotective agents.

Based on previously described data, the aim of this study is to identify and characterize Ocellatin-K1(1–16) and Ocellatin-K1(1–21) peptides isolated from cutaneous secretion of the tropical frog, *Leptodactylus vastus*, as a possible antioxidant agent *in vivo* and *in vitro*.

## Results

### Isolation and structure characterization of new ocellatins

**Figure 1:**
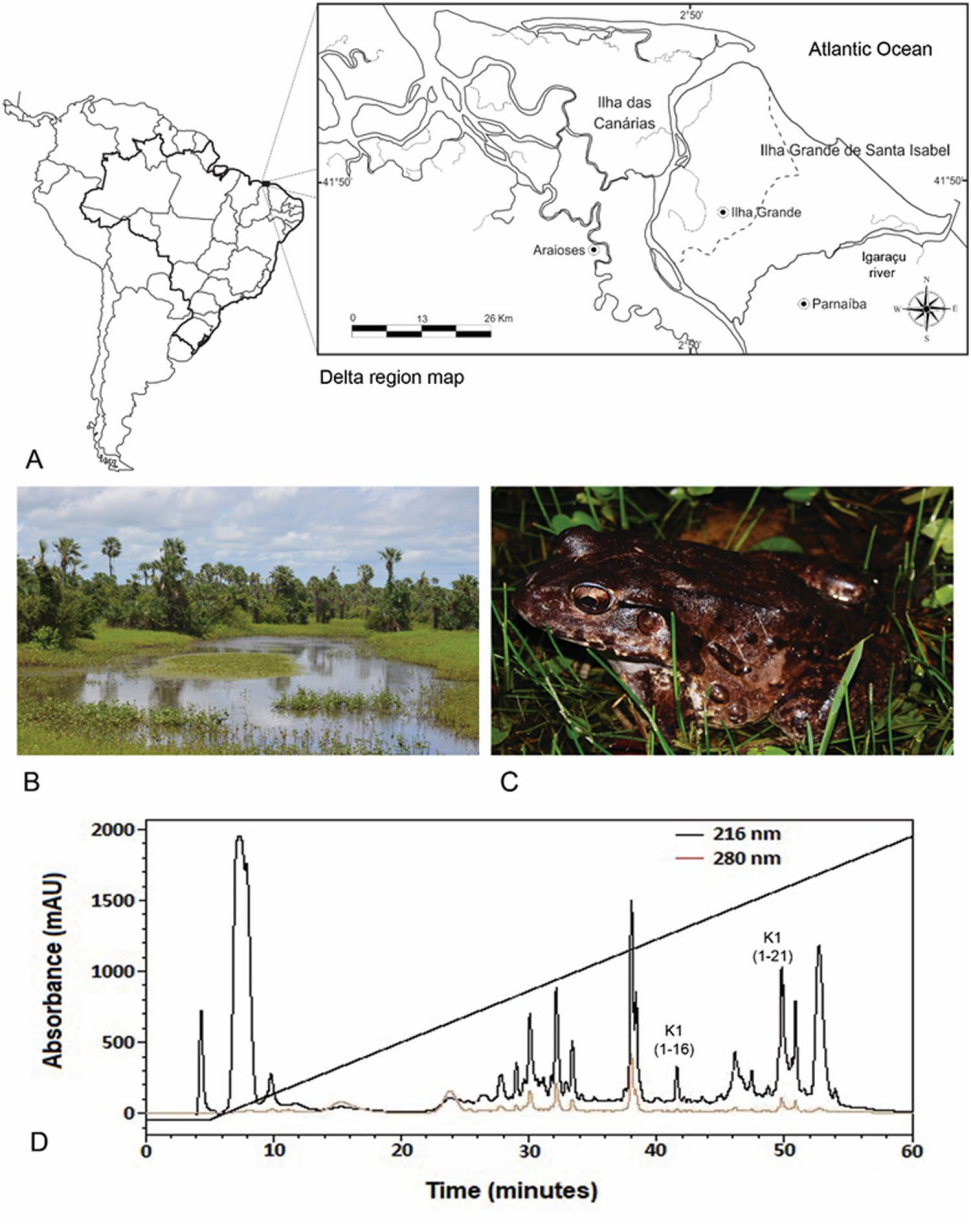
The largest delta in open seas in the Americas and the third largest in the world, the Rio Parnaiba Delta covers 70 islands within its 2,700 km^2^ area, that includes dunes, mangrove forest, and streams (**A**) (ArcMap v10.3, http://desktop.arcgis.com/en/arcmap/10.3). A temporary pond located in the city of Ilha Grande, State of Piaui. This habitat is home to *L. vastus* in this region (**B**). Adult male specimen of *L. vastus* (Photo: Jose Roberto S. A. Leite) (**C**). Reverse-phase HPLC chromatogram of the crude extract from *L. vastus* skin secretion. Fractions containing Ocellatin-K1(1–16) and Ocellatin-K1(1–21) are shown (**D**).

The identified and characterized ocellatins in this study were isolated from the amphibian, *Leptodactylus vastus*, which is found in an ecotonal region of the Brazilian northeast, undergoing great climatic variations, especially during periods of prolonged drought, often leading to mishaps during expeditions and dead individuals near temporary ponds (Fig. 1A–C). The chromatogram obtained from the cutaneous secretion of *L. vastus* presented diverse components absorbing at 216 and 280 nm, suggesting that it contained several potential bioactive peptides (Fig. 1D). The peptide identification strategy of this work was outlined for Ocellatin class molecules. The mass spectrometry analysis performed for chromatographic fractions eluted between 40–50 min revealed two ions with monoisotopic masses of [M + H]^+^ = 1563.9 and [M + H]^+^ = 2062.3 Da. *De novo* sequencing of these ions revealed the structures

**Figure 2:**
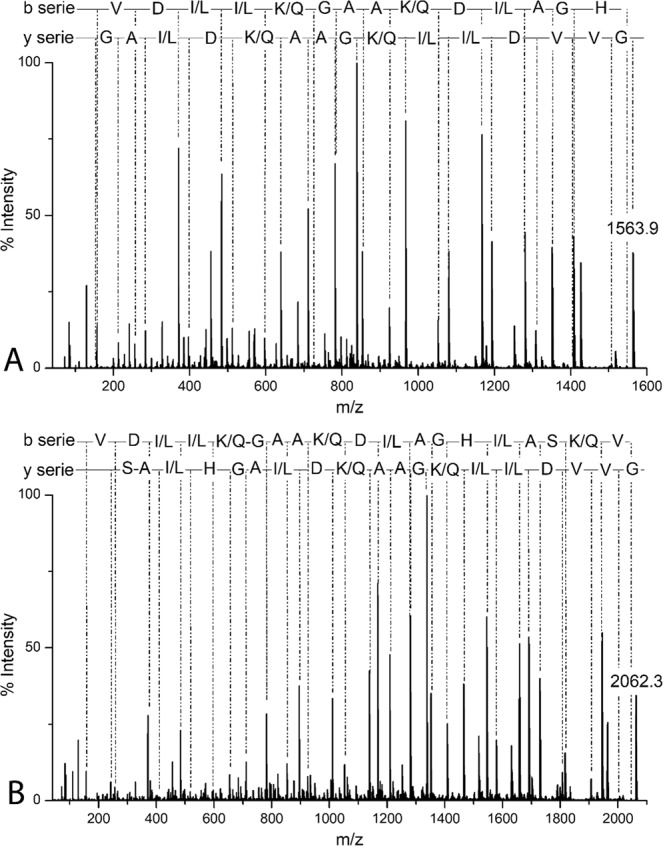
MS/MS spectra for de novo sequencing of Ocellatin-K1(1–16), [M + H] ^±^  = 1563.9 Da (**A**) and Ocellatin-K1(1–21), [M + H]^±^  = 2062.3 Da (**B**) acquired using an MALDI-TOF/TOF UltraFlex Xtreme mass spectrometer.

GVVDI/LI/LK/QGAAK/QDI/LAGH (Fig. 2A) and GVVDI/LI/LK/QGAAK/QDI/LAGHI/LASK/QV (Fig. 2B), respectively. Additionally, the C-terminal of both peptides were not post-translated modified being R-COOH. I/L and K/Q ambiguities were resolved by Edman degradation and the primary structures were confirmed as GVVDILKGAAKDLAGH and GVVDILKGAAKDLAGHLASKV.

A search for similarities shows that both peptides reassembles structurally to ocellatins isolated from skin secretion of *Leptodactylus* frogs (Table 1)^14–22^. The alignment shows that both peptides have structural similarity to Ocellatin-K1 (UniProtKB/Swiss-Prot: P86711.1), comprised of 25 amino acid residues, and isolated from the cutaneous secretion of Amazonian toad-frog *Leptodactylus knudseni*. *L. knudseni*, *L. fallax*, *L. pentadactylus* and *L. vastus* comprise the groups of species named *Leptodactylus pentadactylus*^23^. As both deduced peptides characterized here presented 16 (GVVDILKGAAKDLAGH) or 21 (GVVDILKGAAKDLAGHLASKV) amino acid residues identical to Ocellatin K1, and can be considered as truncated peptides of Ocellatin-K1, they were named Ocellatin-K1(1–16) and Ocellatin-K1(1–21), respectively. In fact, an m/z corresponding to the Ocellatin-K1 was identified but its characterization was not possible because its low intensity signal presented in the mass spectrum (data not showed). Furthermore, the peptides studied in this work are also very closely related to Ocellatin-F, Ocellatin-L1 and Ocellatin-L2, both isolated from cutaneous secretions of amphibians from Central America.

**Table 1:** Sequence, Mw, pI, and hydrophobic moment of ocellatins class peptides isolated from *Leptodactylus* genus.

Name	Sequence																																	Mw^¶^(Da)	pI	µH	Species
Ocellatin-K1 (1–16)	G	V	V	D	I	L	K	G	A	A	K	D	L	A	G	H	—	—	—	—	—	—	—	—	—	—	—	—	—	—	—	—	—	1563.82	6.75	0.483	L. vastus*
Ocellatin-K1 (1–21)	G	V	V	D	I	L	K	G	A	A	K	D	L	A	G	H	L	A	S	K	V	—	—	—	—	—	—	—	—	—	—	—	—	2062.44	8.51	0.399	L. vastus*
Ocellatin-K1	G	V	V	D	I	L	K	G	A	A	K	D	L	A	G	H	L	A	S	K	V	M	N	K	**I**	—	—	—	—	—	—	—	—^a^	2549	9.53	0.256	*L. knudseni-L. vastus*
Ocellatin-F (fallaxin)	G	V	V	D	I	L	K	G	A	A	K	D	**I**	A	G	H	L	A	S	K	V	M	N	K	**L**	—	—	—	—	—	—	—	—^a^	2549	9.53	0.258	*L. fallax/L. pentad-actylus*
Ocellatin-L1 (laticeptin)	G	V	V	D	I	L	K	G	A	A	K	D	L	A	G	H	L	A	**T**	K	V	M	N	K	**L**	—	—	—	—	—	—	—	—	2563.1	9.53	0.244	*L. laticeps*
Ocellatin-L2	G	V	V	D	I	L	K	G	A	A	K	D	L	A	G	H	L	A	**T**	K	V	M	D	K	**L**	—	—	—	—	—	—	—	—	2564	8.44	0.247	*L. laticeps*
Ocellatin-S (syphaxin)	G	V	**L**	D	I	L	K	G	A	A	K	D	L	A	G	H	**V**	A	**T**	K	V	I	N	K	I	—	—	—	—	—	—	—	—^a^	2545	9.53	0.223	*L. syphax*
Ocellatin-V1	G	V	V	D	I	L	K	G	A	**G**	K	D	L	**L**	**A**	H	**A**	**L**	S	K	**L**	S	E	K	**V**	—	—	—	—	—	—	—	—^a^	2633.1	8.44	0.14	*L. validus*
Ocellatin-V2	G	V	L	D	I	L	K	G	A	**G**	K	D	L	**L**	**A**	H	**A**	**L**	S	K	**I**	S	E	K	V	—	—	—	—	—	—	—	—^a^	2576	8.44	0.274	*L. validus*
Ocellatin-V3	G	V	**L**	D	I	L	**T**	G	A	**G**	K	D	L	**L**	**A**	H	**A**	**L**	S	K	**L**	S	E	K	**V**	—	—	—	—	—	—	—	—^a^	2549	6.75	0.295	*L. validus*
Ocellatin-1	G	V	V	D	I	L	K	G	A	**G**	K	D	L	**L**	**A**	H	L	V	G	K	I	S	E	K	**V**	—	—	—	—	—	—	—	—^a^	2560	8.44	0.309	*L. ocellatus*
Ocellatin-2	G	V	**L**	D	I	F	K	D	A	A	K	Q	**I**	**L**	**A**	H	A	A	E	**Q**	**I**	—	—	—	—	—	—	—	—	—	—	—	—^a^	2379.7	6.75	0.269	*L. ocellatus*
Ocellatin-3	G	V	**L**	D	I	L	K	N	A	A	K	N	**I**	**L**	**A**	H	A	A	E	**Q**	**I**	—	—	—	—	—	—	—	—	—	—	—	—^a^	2202.5	6.75	0.438	*L. ocellatus*
Ocellatin-4	G	L	**L**	D	F	V	T	**G**	**V**	**G**	K	D	**I**	F	**A**	**Q**	L	**I**	K	**Q**	**I**	—	—	—	—	—	—	—	—	—	—	—	—^a^	2275.7	5.96	0.473	*L. ocellatus*
Ocellatin-5	G	L	**L**	D	F	L	K	**A**	A	**G**	K	G	L	**V**	T	**N**	**L**	—	—	—	—	—	—	—	—	—	—	—	—	—	—	—	—^a^	1730.0	8.59	0.489	*L. ocellatus*
Ocellatin-6	A	V	**L**	D	F	I	K	**A**	A	**G**	K	G	L	**V**	T	N	**I**	**M**	E	K	V	G	—	—	—	—	—	—	—	—	—	—	—^a^	2274.7	8.54	0.344	*L. ocellatus*
Ocellatin-P (penta-dactylin)	G	L	**L**	D	T	L	K	G	A	A	K	N	V	**Vz**	G	S	L	A	S	K	V	M	E	K	**L**	—	—	—	—	—	—	—	—^a^	2543	9.53	0.324	*L. penta-dactylus*
Ocellatin-PT1	G	V	F	D	I	**I**	K	D	A	**G**	K	Q	L	**V**	**A**	H	**A**	**M**	G	K	**I**	**A**	E	K	**V**	—	—	—	—	—	—	—	—^a^	2639.1	8.44	0.267	*L. pustullatus*
Ocellatin-PT2	G	V	F	D	I	**I**	K	D	A	**G**	K	Q	L	**V**	**A**	H	**A**	T	G	K	**I**	**A**	E	K	**V**	—	—	—	—	—	—	—	—^a^	260.9	8.44	0.272	*L. pustullatus*
Ocellatin-PT3	G	V	**I**	D	I	**I**	K	G	A	**G**	K	D	L	**I**	**A**	H	**A**	**I**	G	K	**L**	**A**	E	K	**V**	—	—	—	—	—	—	—	—^a^	253.0	8.44	0.271	*L. pustullatus*
Ocellatin-PT4	G	V	F	D	I	**I**	K	G	A	**G**	K	Q	L	**I**	**A**	H	A	**M**	G	K	**I**	**A**	E	K	**V**	—	—	—	—	—	—	—	—^a^	2595.1	9.53	0.252	*L. pustullatus*
Ocellatin-PT5	G	V	F	D	I	**I**	K	D	A	**G**	R	Q	L	**V**	**A**	H	A	**M**	G	G	K	**I**	**A**	E	**K**	**V**	—	—	—	—	—	—	—^a^	2667.1	8.5	0.268	*L. pustullatus*
Ocellatin-PT6	G	V	F	D	I	**I**	K	G	A	**G**	K	Q	L	**I**	**A**	H	A	**M**	E	E	K	**I**	**A**	E	K	V	G	L	N	K	D	G	N	3365.9	8.39	0.23	*L. pustullatus*
Ocellatin-PT7	G	V	F	D	I	**I**	K	G	A	**G**	K	Q	L	**I**	**A**	H	A	**M**	G	G	K	**I**	**A**	E	K	V	G	L	N	K	D	G	N	3293.8	9.4	0.212	*L. pustullatus*
Ocellatin-PT8	G	V	F	D	I		K	G	A	**G**	K	Q	L	**I**	**A**	R	A	**M**	G	K	I	**A**	**E**	K	V	G	L	N	K	D	G	N	—	3312.9	9.82	0.214	*L. pustullatus*

^∞^Underline letters represent conservative substitutions.

^a^C -terminus amidated peptide. *Peptides this work.

**Figure 3:**
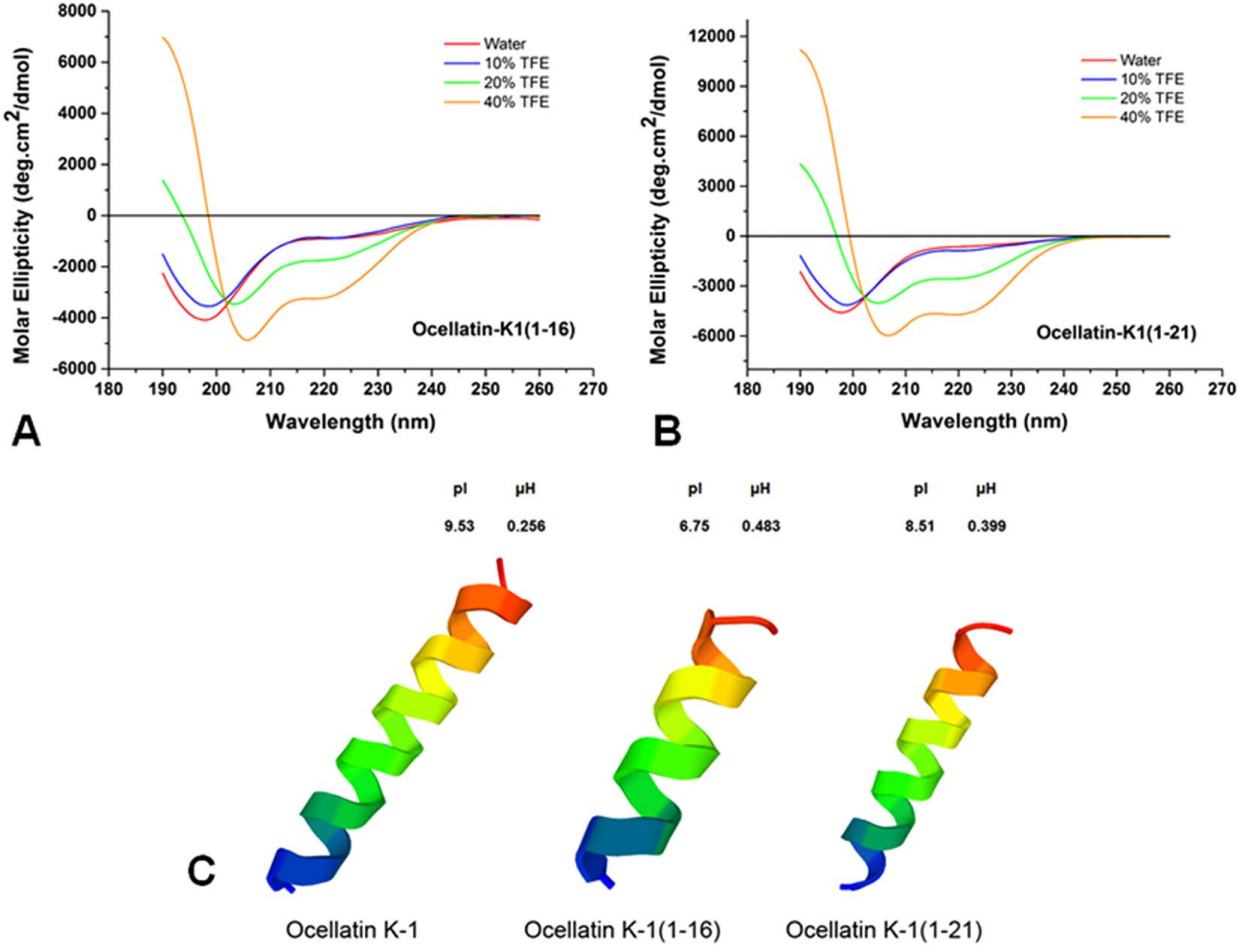
Circular dichroism of peptides in aqueous solution and in 2,2,2-TFE. (**A**) Ocellatin-K1(1–16) and (**B**) Ocellatin-K1(1–21). (**C**) 3D structural model predictions of the ocellatins from *Leptodactylus vastus* (this study) compared to those of Ocellatin-K1 from *Leptodactylus knudseni* (Knudsen’s thin-toed frog).

The results of *in silico* analyses for molecular modeling and experimental data of circular dichroism show that these linear cationic peptides tend to possess an alpha-helix formation in the presence of TFE. In aqueous solution, they are randomized, but in TFE concentrations of 10 to 40%, the formation of secondary structures occurs (Fig. 3).

### Antibacterial activity against strains of *Escherichia coli* and *Staphylococcus aureus*

**Figure 4:**
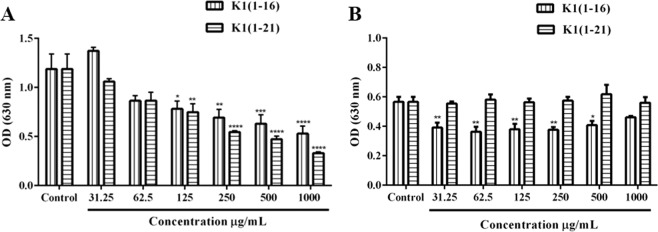
Assessment of anti-microbial activity of Ocellatin-K1(1–16) and Ocellatin-K1(1–21) by MIC assays against *E. coli* (**A**) and *S. aureus* (**B**) strains in a range of concentrations from 31.25 to 1000 μg/mL. The tests were performed in a single assay in triplicate. The results are expressed as mean ± SEM. *p < 0.05 *vs*. control group; **p < 0.01 *vs*. control group; ***p < 0.001 *vs*. control group; **** *p* < 0.0001 *vs*. control group. ANOVA and Sidak test. Abbreviations: K1(1–16): Ocellatin-K1(1–16); K1(1–21): Ocellatin-K1(1–21); OD: optical density.

To analyze the antibacterial activity of Ocellatin-K1(1–16) and Ocellatin-K1(1–21), we assayed the peptides against Gram-negative (*Escherichia coli* ATCC 25922) and Gram-positive (*Staphylococcus aureus* ATCC 25923) strains and MIC values were determined. The results obtained for both the ocellatins showed weak inhibitory activity against *E. coli* with MIC of 125 μg/mL (Fig. 4) and inhibition percentage corresponding to 34.17 ± 11.66%. The optical density (630 nm) of *E. coli* decreased in a dose-dependent manner, showing significant reduction on viability for both the ocellatins at concentrations between 125 and 1000 μg/mL. The value 125 μg/mL of MIC is too high to be characterized as having significant antibacterial potential. Moreover, only Ocellatin-K1(1–16) showed any significant activity against *S. aureus* featuring MICs of 31.25 μg/mL and inhibition percentage corresponding to 30.79 ± 10.27%. This activity was not seen to be concentration dependent. Ocellatin-K1(1–21) did not exert antibacterial activity against *S. aureus* at any of the tested concentrations (31.25 to 1000 μg/mL).

### Effect of Ocellatin-K1(1–16) and Ocellatin-K1(1–21) on superoxide dismutase relative enzymatic activity

**Figure 5:**
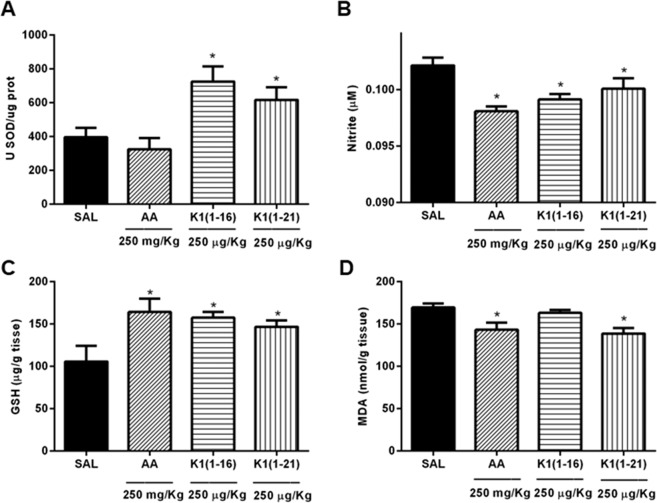
Oxidative parameters in hippocampus of mice acutely treated with Ocellatin-K1(1–16) and Ocellatin-K1(1–21). (**A**) SOD relative enzymatic activity, (**B**) nitrite content, (**C**) GSH, and (**D**) MDA concentration. Ascorbic acid was used as a standard antioxidant. The results are expressed as mean ± SEM of a minimum of six animals per group. *p < 0.05 *vs*. saline group employing ANOVA and Newman–Keuls test. Abbreviations: AA: ascorbic acid; GSH: reduced glutathione; K1(1–16): Ocellatin-K1(1–16); K1(1–21): Ocellatin-K1(1–21); MDA: malondialdehyde; SAL: saline; SOD: superoxide dismutase.

The results showed that SOD relative enzymatic activity in the hippocampal tissue homogenate of animals treated with saline (control) alone was 396.3 ± 54.29 U SOD/µg protein. Acute treatment with Ocellatin-K1(1–16) and Ocellatin-K1(1–21) at the tested doses (250 µg/kg) significantly (*p* < 0.05) stimulated SOD relative enzymatic activity (724.9 ± 88.90 U SOD/µg protein; and 616.0 ± 75.61 U SOD/µg protein, respectively) compared to those treated with saline. Ascorbic acid (250 mg/kg), an antioxidant standard, demonstrated basal levels of the endogenous antioxidant SOD (324.3 ± 66.61 U SOD/µg protein) compared to those of control as shown in Fig. 5A.

### Effect of Ocellatin-K1(1–16) and Ocellatin-K1(1–21) on nitrite reduction

Acute administration of Ocellatin-K1(1–16) and Ocellatin-K1(1–21) significantly reduced (*p* < 0.05) the basal content of nitrite (0.0991 ± 0.0004 µM; and 0.1001 ± 0.0009 µM, respectively) compared to those observed in the saline group (0.1021 ± 0.0007 µM). This result suggests that treatment with ocellatins protects the hippocampus. Furthermore, ascorbic acid significantly reduced (*p* < 0.05) the nitrite content (0.0980 ± 0.0004 µM) compared with that of the control group (Fig. 5B). Thus, mice that were not treated with either ocellatins or ascorbic acid had higher contents of nitrite.

### Effect of Ocellatin-K1(1–16) and Ocellatin-K1(1–21) on glutathione concentration

Acute administration of both Ocellatin-K1(1–16) and Ocellatin-K1(1–21), cellular defence agents, significantly elevated (*p* < 0.05) the GSH concentration (157.4 ± 6.71 µg/g tissue and 146.6 ± 7.62 µg/g tissue, respectively) compared to those with saline group (105.6 ± 18.68 µg/g tissue). Therefore, the protective effect of ocellatins administration might be explained by a resultant increase in the hippocampal GSH concentration. Administration of ascorbic acid (164.1 ± 15.60 µg/g tissue) also elevated hippocampal GSH concentration in the experimental group when compared to those in the saline group, as shown Fig. 5C.

### Effect of Ocellatin-K1(1–16) and Ocellatin-K1(1–21) on malondialdehyde concentration

Our results suggest that the administration of Ocellatin-K1(1–16) peptide maintained the basal concentration of MDA (163.0 ± 3.31 nmol/g tissue) and did not result in a significant difference in MDA concentration in the mice hippocampi when compared with those in saline group (169.5 ± 4.84 nmol/g tissue) as shown the Fig. 5D. However, Ocellatin-K1(1–21) peptide significantly (*p* < 0.05) reduced the redox state of the hippocampi of animals in the experimental group to 138.6 ± 6.61 nmol/g tissue when compared with animals treated with saline. Furthermore, the ascorbic acid (143.1 ± 8.53 nmol/g tissue) treatment also showed a significant (*p* < 0.05) effect compared to that of control group.

### Effect of Ocellatin-K1(1–16) and Ocellatin-K1(1–21) on LPS-induced NF-kB activation in microglia

**Figure 6:**
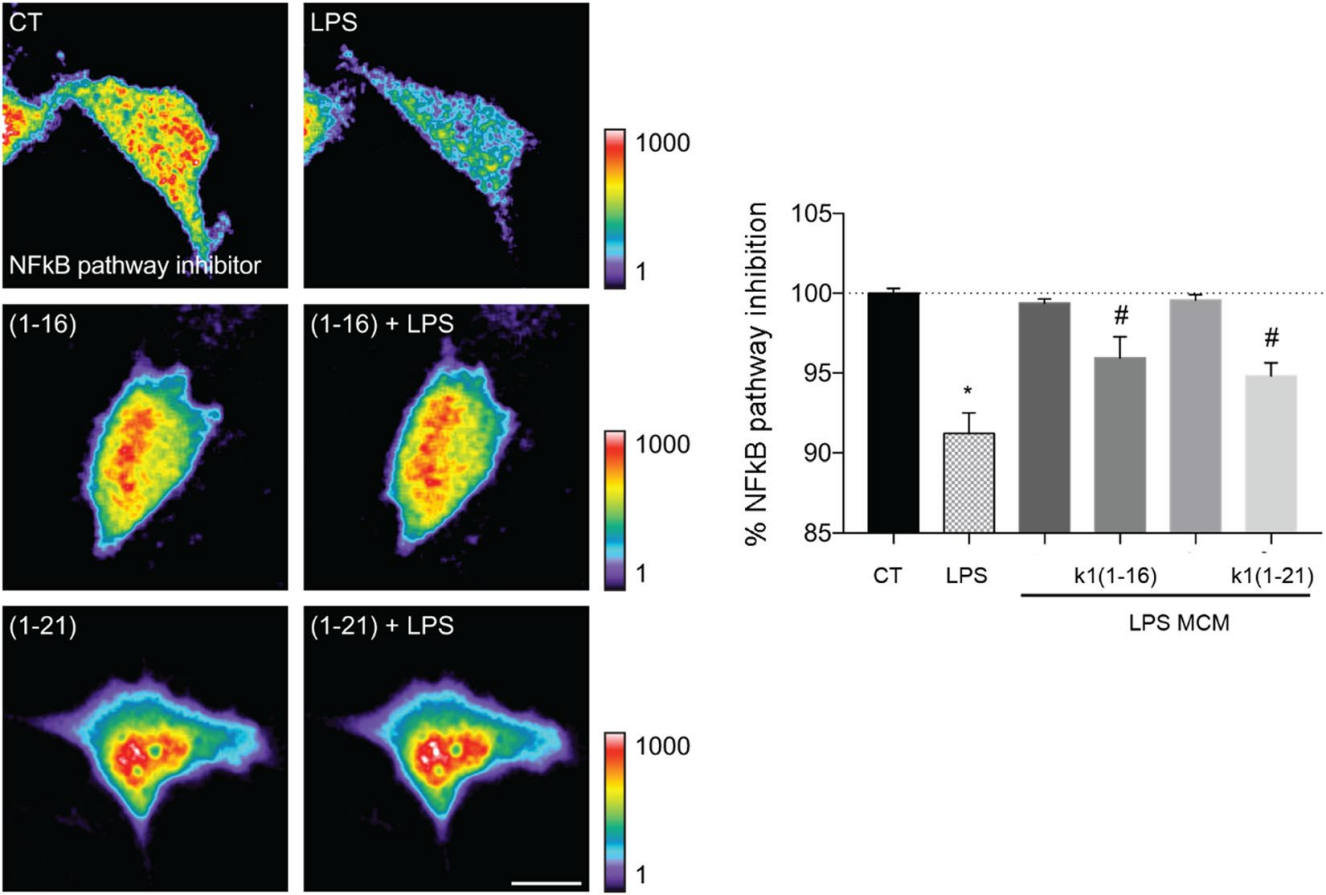
Ocellatin-K1(1–16) and Ocellatin-K1(1–21) prevented the LPS-induced NF-kB activation in living microglia. CHME3 human microglial cells expressing the biosensor of NF-kB pathway inhibitor were incubated with 100 μM Ocellatin-K1(1–16) or Ocellatin-K1(1–21) and challenged with 1 μg/mL LPS. Microglia were incubated only with Ocellatin-K1(1–16) and Ocellatin-K1(1–21) and compared with saline control group. Time-lapse fluorescence intensities for the NF-kB pathway inhibitor biosensor are shown (n = 17–19 cells pooled across two different experiments). The results are expressed as mean ± SEM. ^#^*p* < 0.01 *vs*. LPS group; *p < 0.001 *vs*. CT not treated with LPS employing one-way ANOVA with Bonferroni post-test. Abbreviations: CT: control; LPS: lipopolysaccharide.

As demonstrated above, ocellatins decreased the basal ROS production and the oxidative state of the mice hippocampi. For a better understanding the role of the ocellatins redox regulation, we analysed the Ocellatin-K1(1–16) and Ocellatin-K1(1–21) effect on lipopolysaccharide (LPS)-induced NF-kB activation in living microglia using time-lapse video microscopy. To achieve this, we transfected the microglial cells with the biosensor of NF-kB pathway inhibitor and observed that incubation with 100 μM of either Ocellatin-K1(1–16) or Ocellatin-K1(1–21) significantly (*p* < 0.01) prevented LPS-induced NF-kB activation in microglia when compared with LPS only-treated group (Fig. 6) with no change in NF-kB basal activity.

### Effect of Ocellatin-K1(1–16) and Ocellatin-K1(1–21) on microglial-induced ROS formation in hippocampal neurons

**Figure 7:**
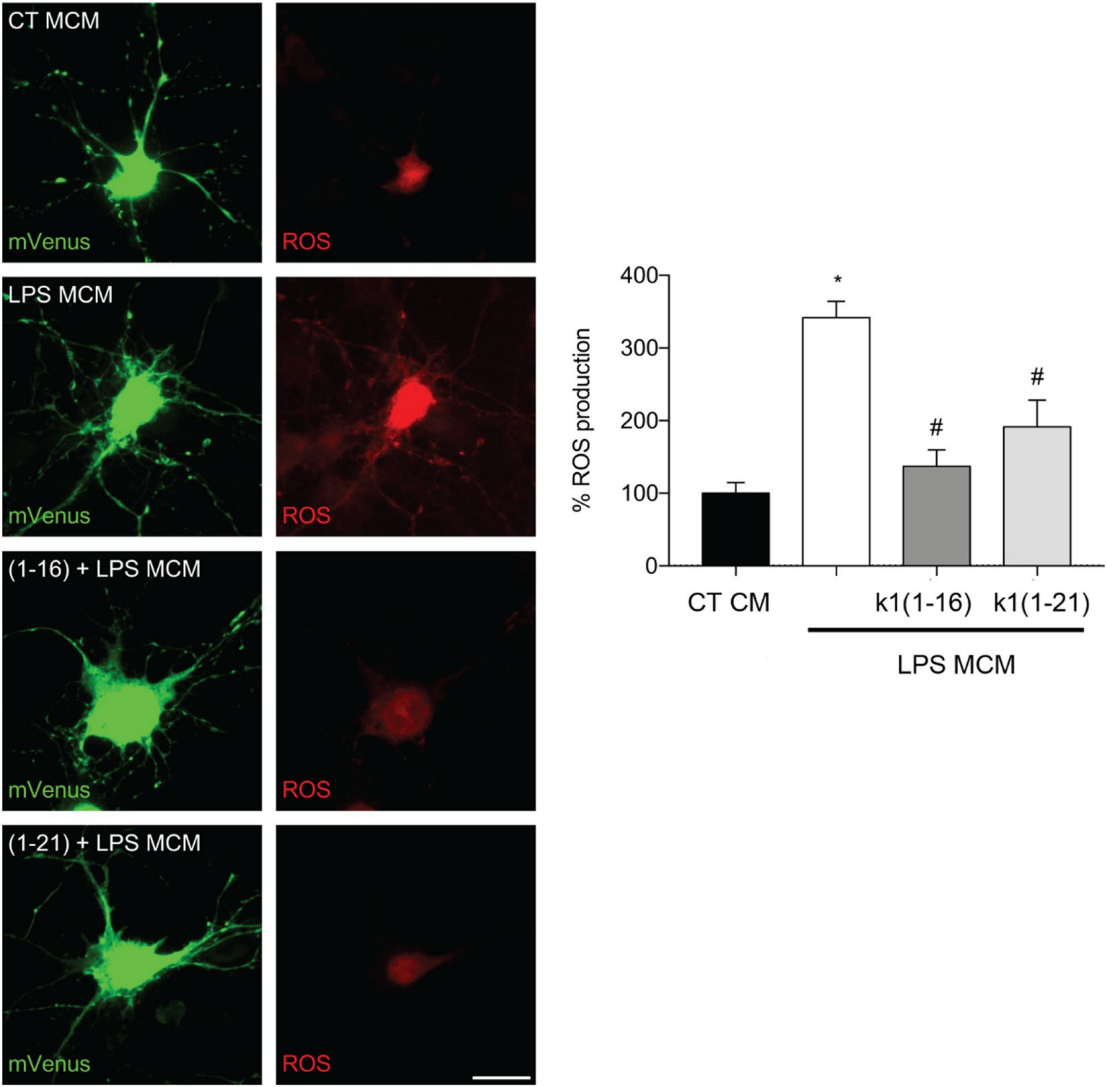
Ocellatin-K1(1–16) and Ocellatin-K1(1–21) protect hippocampal neurons from oxidative stress induced by LPS-treated-microglial conditioned media. Representative confocal images and quantification of ROS production in hippocampal neurons incubated with conditioned medium from microglia subjected to LPS-induced and 100 μM Ocellatin-K1(1–16) or Ocellatin-K1(1–21). Images show neurons expressing mVenus (green) and the Hyper Red ROS biosensor (red). The results are expressed as mean ± SEM calculated from 3 different cultures. *p < 0.001 *vs*. LPS group; ^#^*p* < 0.001 *vs*. CT employing one-way ANOVA with the Bonferroni post-test. Abbreviations: CT: control; LPS: lipopolysaccharide; MCM: microglia conditioned medium.

The overactivated microglia released several molecules that could induce neuronal damage^24^. Since we observed that Ocellatin-K1(1–16) and Ocellatin-K1(1–21) blocked the LPS-induced activation of NF-kB by microglia, we hypothesized that ocellatins can protect hippocampal neurons from oxidative stress induced by microglial activation. Towards this objective, we treated hippocampal neurons with microglial conditioned medium, and observed a significant (*p* < 0.001) increase in ROS formation induced by the conditioned media obtained from LPS-treated microglia in comparison with conditioned media from control microglia (Fig. 7). We observed that both Ocellatin-K1(1–16) and Ocellatin-K1(1–21) significantly (*p* < 0.001) reduced neuronal oxidative stress elicited by LPS-treated microglia.

### Haemolytic Assays of Ocellatin-K1(1–16) and Ocellatin-K1(1–21)

**Figure 8:**
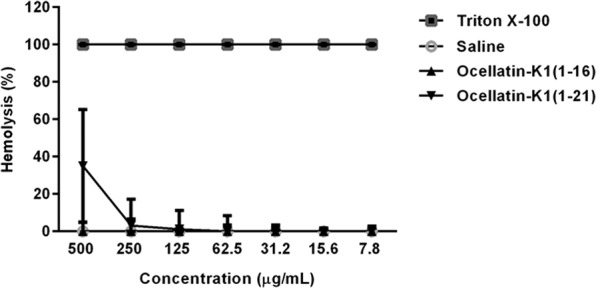
Haemolytic activity of Ocellatin-K1(1–16) and Ocellatin-K1(1–21) in human erythrocytes at concentrations ranging from 7.8 to 500 µg/mL. A positive control was determined using a 10% solution of Triton X-100. ANOVA and *t*-test were used for statistical analysis.

The haemolytic activities of ocellatins against human erythrocytes, as summarized in Fig. 8, indicate that Ocellatin-K1(1–16) peptide did not give rise to lysis at the concentrations tested (7.8 to 500 µg/mL), and there was no significant difference between the effects of ocellatins and the negative control (saline). However, the highest concentration (500 μg/mL) of the Ocellatin-K1(1–21) analysed, demonstrated approximately 35% haemolysis. Thus, these results suggest that Ocellatin-K1(1–16) is less toxic than Ocellatin-K1(1–21) in lysing erythrocytes.

### Effect of Ocellatin-K1(1–16) and Ocellatin-K1(1–21) on erythrocyte morphology and roughness

**Figure 9:**
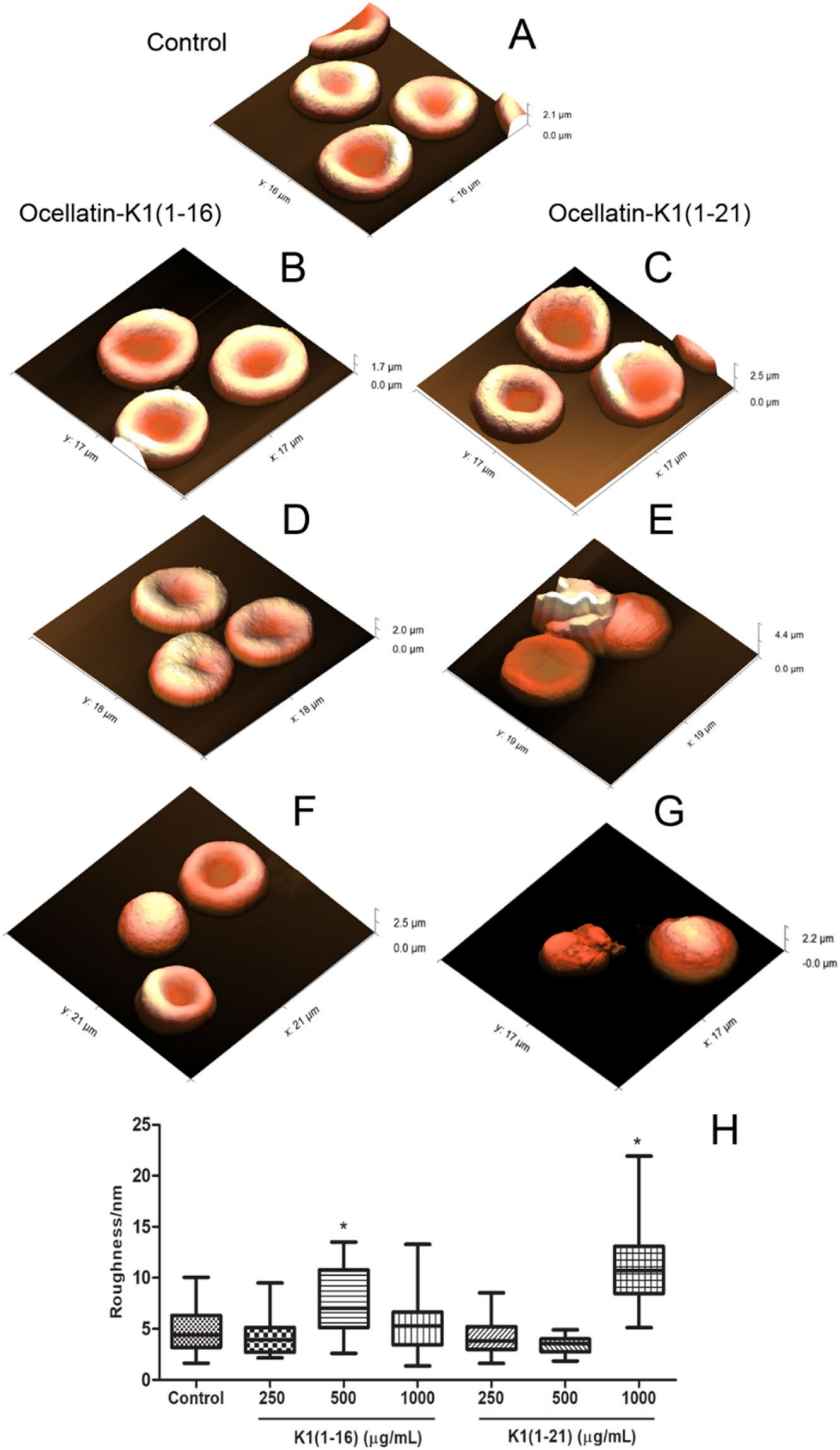
AFM representative images showing the morphology and roughness of human erythrocytes. (**A**) Morphology of untreated erythrocytes (control group) and after exposure to the Ocellatin-K1(1–16) (**B**,**D**,**F** at 250, 500, and 1000 µg/mL, respectively) and to the Ocellatin-K1(1–21) (**C**,**E**,**G** at 250, 500, and 1000 µg/mL, respectively). (**H**) Average roughness of human erythrocytes untreated (control) and treated with Ocellatin-K1(1–16) and Ocellatin-K1(1–21) at concentrations ranging from 250 to 1000 µg/mL. *p < 0.0001 *vs*. control group using ANOVA and *t*-test. Abbreviations: K1(1–16): Ocellatin-K1(1–16); K1(1–21): Ocellatin-K1(1–21).

As demonstrated above, ocellatins showed low haemolytic activity in human erythrocytes. Thus, we analysed the human erythrocyte membrane morphology and roughness under AFM and the representative results are shown in Fig. 9. Untreated erythrocytes appeared as typical biconcave shape (Fig. 9A). After 30 minutes of incubation with Ocellatin-K1(1–16), no significant morphological changes in the cells were found at the concentrations tested (250, 500 and 1000 µg/mL; Fig. 9B–F, respectively). However, erythrocytes treated with Ocellatin-K1(1–16) peptide at a concentration 500 µg/mL demonstrated a significant increase of roughness (7.64 ± 0.56 nm) compared to that observed in untreated samples (4.68 ± 0.22 nm), and this finding was statistically (*p* < 0.0001) significant (Fig. 9H). In addition, it was observed that the biconcave topography of erythrocytes was altered by incubation with Ocellatin-K1(1–21) at a concentration 500 µg/mL for 30 minutes (Fig. 9E). Thus, characteristic protrusions indicating damage to the erythrocyte membrane were observed, which were not seen in control cells. Furthermore, exposure to Ocellatin-K1(1–21) at a concentration of 1000 µg/mL resulted in a significant change in the cell shapes (Fig. 9G) compared to that in control group. This damage could be confirmed by the significant increase in the membrane roughness (11.44 ± 0.74 nm) of erythrocytes treated with Ocellatin-K1(1–21) at concentration of 1000 µg/mL, which was significantly increased (*p < *0.0001) as compared to that in control cells.

## Discussion

Amphibian’s skins are a rich resource of peptides, which have diverse biological activities and are regarded as potential sources of new therapeutic agents^25^. Studies have focused on the purification of novel antimicrobial peptides due to their considerable association with innate defence mechanisms^26^. In the current study, we analysed the antioxidant activity of two peptides purified from skin secretions of *L. vastus*. According to the sequence alignment of the structures obtained, the peptides were designated as Ocellatin-K1(1–16) and Ocellatin-K1(1–21) because they were leptodactylid peptides with similarities in amino acid sequence to the ocellatins. Many studies involving characterization of leptodactylid antimicrobial peptides have also been described^18^. However, Ocellatin-K1(1–16) and Ocellatin-K1(1–21) showed weak antibacterial activity against the *E. coli* ATCC 25922 and *S. aureus* ATCC 25923 strains tested. Previous reports indicate that not all ocellatins have antibacterial activity. Studies with *Leptodactylus pustulatus*^22^ revealed that Ocellatin-PT2 did not inhibit Gram-negative bacterial strains; however, Ocellatin-PT7 and -PT8 demonstrated antibacterial activities against a Gram-positive strain with low antimicrobial potency. In this study, other homologous ocellatins, such as ocellatin-PT1, -PT3, -PT4, -PT5 and -PT6 inhibited one or more Gram-negative and -positive bacterial strains. These peptides differ by only a few amino acid substitutions and present different bactericidal activities. In our study, employing sequence alignments, Ocellatin-K1(1–16) and Ocellatin-K1(1–21) peptides showed sequence similarities with such ocellatins obtained from *L. pustulatus*. Thus, it may be suggested that small differences in sequences can lead to important differences in the activity spectra of the peptides. In addition, the antimicrobial activity of peptides is determined by a set of factors, such as conformation, net charge, hydrophobicity, and amphipathicity^27^. Further, despite Ocellatin-K1(1–16) and Ocellatin-K1(1–21) tend to adopt α-helices conformation at a hydrophobic environment, a trait of amphibian’s antimicrobial peptide, they are truncated peptides of Ocellatin-K1 that possess the “additional” motif MNKL-NH_2_, when compared to Ocellatin-K1(1–21). The absence of the referred motif, as well as the lack of C-terminal amidation, may be promoting the reduced antimicrobial activities observed for Ocellatin-K1(1–16) and Ocellatin-K1(1–21). C-terminal amidation is directly associated to an improvement in the abilities of cationic peptides to interact with biological membranes, a prerequisite to action of membrane active antimicrobial peptides^28–30^. Finally, the study of truncated peptides was reported for *Hypsiboas raniceps* and was associated to the loss of antimicrobial activity when compared to the intact molecule, probably reflecting distinct protective role between stored and secreted peptides^31^.

Considering that anura of *L. vastus* species live in the Parnaiba Delta Region, Northeast of Brazil, with exposure to strong and long periods of sunlight radiation, wherein their skins are exposed to elevated ultraviolet radiation, they are likely to possess a specific and highly effective antioxidant system. Thus, the main objective of this study was to test if the new peptides isolated from cutaneous secretion of *L. vastus* have antioxidant activity. *In vivo* studies were performed using isolated hippocampus due to its high sensitivity to oxidative stress. Neural tissue has a high rate of oxygen consumption and possesses a high metabolic activity, and therefore, is a tissue more vulnerable to lipid peroxidation as compared to other tissues^32^. Consequently, antioxidant peptides, which rapidly exert their biological functions, have a potential to prevent neuronal damage related to lipid peroxidation. Ocellatin-K1(1–16) and Ocellatin-K1(1–21) have small structures that help penetrate the blood–brain barrier (BBB) and exert antioxidant effects in the hippocampus. Indeed, some peptides cross the BBB through endocytic mechanisms involving receptor mediated transcytosis and/or adsorptive-mediated transcytosis. In recent years, BBB shuttle peptides have received growing attention because of their lower cost, reduced immunogenicity, biologic specificity and higher chemical versatility^33^.

We demonstrated that Ocellatin-K1(1–16) and Ocellatin-K1(1–21) protected against oxidative stress at the concentrations tested. Our ocellatins were effective in increasing SOD activity and the basal concentration of GSH in hippocampal tissues. These markers protect cells against deleterious effects of free oxygen radicals, thus providing a defence mechanism for the survival of aerobic organisms. SOD, a metalloenzyme, catalyses the dismutation of superoxide anions into oxygen and hydrogen peroxide. In addition, the tripeptide, GSH, protects cells against damage caused by reactive oxygen species, including free radicals and peroxides^34^. This study shows that the ocellatins decrease basal nitrite content, a nitric oxide metabolite, thus corroborating with the results cited above for SOD since this enzyme reduces nitrite levels by up to 50%^35^. Ocellatin-K1(1–16) only maintained basal concentration of MDA, a marker of oxidative stress and lipid peroxidation product; however, Ocellatin-K1(1–21) significantly reduced concentration of this aldehyde. In view of this observation, it may be suggested that Ocellatin-K1(1–21) has a greater protective effect by acting as a potential antioxidant than that of Ocellatin-K1(1–16). Thus, our findings suggest that exploring the antioxidant effects of the studied ocellatins could be of therapeutic interest in the context of pathologies associated with deficiencies in the enzymatic defence system against oxidative stress. The rapid antioxidant mechanism of peptides extracted from secretions from the skin of amphibians is unknown^2^. However, studies indicated that the presence of proline, methionine, cysteine, tyrosine, or tryptophan residues may contribute to the antioxidant function of peptides^3^. In contrast, it has been reported that peptides extracted from the secretion of amphibians containing the amino acid residues listed above did not necessarily demonstrate antioxidant activity^36^. Interestingly, the ocellatins studied here do not possess any of these amino acid residues.

As we previously described that ocellatins decrease ROS on mice hippocampi, we analysed the effect of Ocellatin-K1(1–16) and Ocellatin-K1(1–21) on LPS-induced NF-kB activation. Experimental studies show that stimulation by LPS induces microglial activation^37^. When microglia activated, they release several factors, such as ROS, and are capable of activate NF-kB transcription factor. NF-kB is a key transcription factor that plays an important role in the microglia activation by expressing iNOS, COX-2, and pro-inflammatory cytokines such as TNF-α, IL-1β, and IL-6^38^. The activation of this transcription factor in microglia is widely associated with dysregulation of normal neuroinflammatory responses that characterizes neurodegenerative diseases^39^. In this study, we showed that Ocellatin-K1(1–16) and Ocellatin-K1(1–21) significantly prevented the LPS-induced NF-kB activation. Considering that the redox state controls NF-kB nuclear levels^38^, these results suggest that inhibition of NF-kB likely contributed to the antioxidant and anti-neuroinflammatory effects of the ocellatins in LPS-stimulated microglial cells. In microglial cells, antioxidants, such as ascorbate^40^ and piperlongumine^41^, also exert their effects by suppressing NF-kB activity.

Proinflammatory activation can induce microglia to release factors that damage neurons^24^. Thus, it is essential to control microglial reactivity in order to protect the neurons from excess ROS. Accordingly, we demonstrated that Ocellatin-K1(1–16) and Ocellatin-K1(1–21) reduced oxidative stress in hippocampal neurons incubated with LPS-treated microglia conditioned media. This suggests that these ocellatins can prevent microglial-induced neuronal toxicity.

The search for new molecules that have promising biological activities and do not cause harmful effects to humans is widely associated with trials investigating the possibility of natural products to injure plasma membranes of human erythrocytes^42^. Therefore, an assay to estimate *in vitro* haemolytic capacity was performed as a toxin- screening method to estimate the potential of Ocellatin-K1(1–16) and Ocellatin-K1(1–21) to cause cell damage that may be induced *in vivo*. Ocellatin-K1(1–16) did not cause haemolytic activity at the concentrations tested in this study, whereas Ocellatin-K1(1–21) demonstrated approximately 35% haemolysis at the highest concentration analysed. These results are consistent with results previously reported^22^, which demonstrated that others ocellatins isolated from the skin secretions of the frog, *L. pustulatus*, have low to none haemolytic activity. Thus, Ocellatin-K1(1–16) and Ocellatin-K1(1–21) showed considerable cellular biocompatibility towards mammalian cells *in vitro*.

Erythrocyte deformability was confirmed by AFM studies, which combines high-resolution imaging and functionality in a physiological environment^43^. Our morphological study on erythrocyte membrane surface indicates that surface irregularities typical of normal erythrocytes were modified following high concentrations of exposure to Ocellatin-K1(1–16) and Ocellatin-K1(1–21). These results were quantitatively described by the morphological roughness parameter of the erythrocyte outer leaflet membranes when comparing surface topographic features on a nanoscale level. These results showed that for both peptides, the concentration of 250 µg/mL, there was no significant change in cell morphology or membrane roughness. For both peptides, this concentration is above that tested in the oxidative stress assays (i.e. more than 100 µM). Only at higher concentrations (500 µg/mL and above), were significant changes seen in membrane texture or cell shape. The changes in the cell morphology of erythrocytes were broadly compatible with the haemolytic tests carried out *in vitro*. Thus, the observations associated with the functional properties of Ocellatin-K1(1–16) and Ocellatin-K1(1–21) suggest possible therapeutic applications.

The results obtained in this study suggest that the Ocellatin-K1(1–16) and Ocellatin-K1(1–21) extracted from cutaneous secretions of the tropical frog, *L. vastus*, possess antioxidant activity in mice hippocampi by increasing SOD activity and GSH concentration, as well as by reducing nitrite content and MDA concentration. In addition, these ocellatins were effective in impairing LPS-induced NF-kB activation in living microglia cells and reducing oxidative stress elicited in hippocampal neurons incubated with conditioned media from LPS-treated microglia. These observations suggest that ocellatins can form the basis for discovery and development of novel agents that might control ROS production and microglial activation in order to treat or prevent oxidative stress associated to several neurological diseases.

## Methods

### Isolation and characterization of the peptides

Adults frogs of *Leptodactylus vastus* (*L. vastus*) Lutz 1930 (North-eastern Pepper Frog) species were manually captured in the Parnaiba Delta Region, Ilha Grande de Santa Isabel, Ilha Grande city, Piaui state, Brazil, under the license number, 61838–1 SISBIO/ICMBio (Fig. 1A–C). The cutaneous secretion from *L. vastus* was obtained by electrical stimulation (9 V), collected in 50 mL tubes containing Milli-Q water, filtered (Millipore filters, 0.22 μm), immediate frozen, and lyophilized.

The lyophilized secretion of *L. vastus* (1 mg) was dissolved in Milli-Q water (500 μL) and subjected to reversed-phase chromatography (Shimadzu Co., LC-20 CE, Kyoyo, Japan), using a Vydac C18 reversed phase column (2018 TP). The fractions were eluted with a linear gradient of 0.1% (v/v) (trifluoroacetic acid) TFA/acetonitrile ranging from 5% to 60% over 60 min and 75–95% over 5 min at a flow rate of 1 mL/min. Fractions were monitored at 216 and 280 nm, collected in tubes, and dried under centrifugation at low pressure.

Chromatographic fractions were dissolved using Milli-Q water (in 100 to 500 µL) according to absorbance obtained. Each sample was mixed with α-cyano-4-hydroxyxinnamic acid (10 mg/mL dissolved in 50% acetonitrile/0.3% TFA (v/v) in Milli-Q water) in a proportion of 1:3 (v/v), spotted onto a MALDI plate and evaporated at room temperature. The samples were analysed using an UltraFlex Xtreme mass spectrometer (Bruker Daltonics, Bilerica, MA) operating in the positive reflective mode. The mass range analysed was between m/z 800 and 5000. Ions were selected for fragmentation using the LIFT™ mode. The mass spectrometer was controlled by FlexControl 4.0 software and MS/MS spectra were manually interpreted using FlexAnalysis 3.4 software. Before acquisition, the mass spectrometer was calibrated using peptide calibration Standard II solution. Further purification steps enabled the confirmation of their amino acid sequences by Edman degradation.

### Peptide synthesis

Ocellatin-K1(1–16), sequence GVVDILKGAAKDLAGH-OH, and Ocellatin-K1 (1–21), sequence GVVDILKGAAKDLAGHLASKV-OH were manually synthesized by the 9-fluorenylmethyloxycarbonyl (Fmoc) solid-phase strategy starting with a Fmoc-His(Trt)-Wang resin (0.42 mmol/g) or with a Fmoc-Val-Wang resin (0.53 mmol/g) at 0.4 mmol scale. Couplings were performed with HBTU:DIPEA (O-(benzotriazol-1-yl)-1,1,3,3-tetramethyluronium hexafluorophosphate:N-ethyl-N-propan-2-ylpropan-2-amine) in N,N-dimethylformamide (DMF) for 60–90 minutes. Fmoc deprotections were accomplished by a 25% 4-methylpiperidine solution in DMF (2 × 15 min). The peptidyl-resin was washed with 2-propanol and DMF (4x each, alternating the solvents) after each coupling and deprotection step. Side chain deprotection and peptide cleavage step was carried out with 15 mL trifluoroacetic acid in the presence of 500 µL water and 500 µL triisopropylsilane. Reagents used for peptide synthesis were acquired from Peptides International (Louisville, KY, USA). Purification was performed on a preparative reversed-phase column (Vydac 218 TP 510, Hesperia, CA) coupled to High Performance Liquid Chromatography (HPLC) system model LC-10VP (Shimadzu Corp., Kyoto City, Japan). The molecular weights and purity of samples were confirmed using MALDI-TOF/TOF mass spectrometry (Ultraflex, Bruker Daltonics, Bilerica, MA), as described elsewhere^44^.

### Structural studies

Three-dimensional structural model predictions of the peptides were obtained using the internet resource, PEP-FOLD 3.5 (*de novo* peptide structure prediction). The secondary structures of the peptides were assessed by circular dichroism (CD) spectroscopy in the far ultraviolet spectrum, using a Jasco J-815 CD Spectropolarimeter (JASCO, Tokyo, Japan) as previously reported^45^. Briefly, the measurements were carried out under a nitrogen gas flow of 8 L/h at 20 °C. Spectra were obtained between 190 and 260 nm. The peptides were used at a concentration of 100 μM in Milli-Q water and at various concentrations in 2,2,2-trifluoroethanol (TFE). These experiments were performed at 37 °C and a scan speed of 50 nm/min; a response time of 1 s and a bandwidth of 1 nm were used. The spectra were converted to molar ellipticity per residue as previously reported^45,46^.

### Antibacterial activity

The bacterial strains *Escherichia coli* ATCC 25922 and *Staphylococcus aureus* ATCC 25923, were used for the determination of antibacterial activity of Ocellatin-K1(1–16) and Ocellatin-K1(1–21) peptides. The bacterial strains were grown at 37 °C in Mueller-Hinton broth until a logarithmic phase was reached [1–2 × 10^8^ colony forming units (CFU)/mL]. Ocellatin-K1(1–16) and Ocellatin-K1(1–21) concentrations varying from 31.25 to 1000 μg/mL were prepared using serial dilutions. In each well, the final inoculum concentration was 5 × 10^5^ CFU/mL. Minimum inhibitory concentration (MIC) was defined as the lowest peptide concentration that inhibited the visible bacterial growth for 24 h at 37 °C, in aerobic conditions. Besides the reading of visible bacterial growth, the optical density (630 nm) was evaluated for the calculation of the percentage of inhibition of bacterial growth where optical density of the untreated bacteria was considered 100% growth. The MIC determination was performed in triplicate and in a single experiment, according to the Clinical & Laboratory Standards Institute (CLSI) protocol^47^.

### Neurochemical studies in mice hippocampus for evaluation of antioxidant status

#### Animals

Male Swiss albino mice (*Mus musculus*), weighing 25–30 g, were maintained at a constant temperature of 25 ± 1 °C. The mice were accommodated in opaque plastic polypropylene cages (maximum six animals per cage) under a standard environment of 12 h light/dark cycle and allowed free access to food and water. Procedures related to the care and use of animals in our experiments were performed in accordance to the Brazilian Society of Sciences of Laboratory Animals. The experiments were previously submitted to approval of the Ethics Committee on Animal Use (CEUA/UFPI) from Federal University of Piaui (protocol no. 001/2019). Euthanasia of animals were performed following the guidelines of the practice of euthanasia of CONCEA (National Council of the Control of Animal Experimentation), normative resolution no. 37 of February 15, 2018.

#### Experimental design

The animals were randomly allocated to four groups, and each group consisted of six animals. Animals in the first group were intraperitoneally (*i.p*.) treated with saline (vehicle) and served as negative control group. The second group received ascorbic acid, a standard antioxidant agent, at a dose of 250 mg/kg. The other two groups were treated with Ocellatin-K1(1–16) and Ocellatin-K1(1–21) at doses of 250 µg/kg. All the administrations were acute, by a single intraperitoneal injection on a single day, according to a previously described method^35^. After 1 h of each intraperitoneal administration, the mice were euthanized by an overdose of xylazine, *N*-(2,6-dimethylphenyl)-5,6-dihydro-4H-1,3-thiazin-2-amine (18 mg/kg) and ketamine, 2-(2-chlorophenyl)-2-(methylamino)cyclohexan-1-one (240 mg/kg). Next, the brains were quickly removed and placed on ice. After dissection, each hippocampus was identified, weighed, and stored at -80 °C for subsequent preparation of homogenates and for biochemical analysis. The hippocampus tissues were used to determine superoxide dismutase (SOD) relative enzymatic activity and reduced glutathione (GSH) concentration, as well as nitrite content and malondialdehyde (MDA) formation.

#### Determination of superoxide dismutase relative enzymatic activity

The SOD relative enzymatic activity were measured by the method described previously^48^. In this method, the enzyme activity is calculated by measuring the amount of SOD capable of inhibiting nitrite formation by 50%. Hippocampus tissues were homogenized in potassium phosphate buffer (50 mM, pH 7,4) to prepare 10% homogenates. Briefly, aliquots of 100 μL of each homogenate was added to 1.11 mL of phosphate buffer, 75 μL of L-methionine (20 mM), 40 μL of Triton X-100 (1% v/v), 75 μL of hydroxylamine hydrochloride (10 mM), and 100 μL of EDTA (50 μM). Next, this mixture was heated in a boiling water bath at 37 °C for 5 min. Further, 80 μL of riboflavin solution (50 μM) were added and the mixture was exposed to light for 10 min. Later, 100 μL of this preparation together with 100 μL of Griess reagent were placed into wells, and the absorbance was measured at 550 nm after 10 min. In addition, the amount of total proteins was estimated using a protein assay kit (Labtest). The SOD relative enzymatic activity were expressed as unit enzyme activity per microgram of protein.

#### Determination of nitrite reduction

Reduction of nitrite was estimated based on the Griess reaction, according to the method described^49^. The hippocampus tissue samples were homogenized in 0.15 M KCl (1 mL/100 mg tissue), under cooling. Next, 100 μL of the supernatant were mixed with 100 μl of Griess reagent at room temperature for 10 min. Absorbance was measured using a microplate reader at 540 nm. Nitrite concentration in the sample was determined using a sodium nitrite (NaNO_2_) standard curve. Results were expressed as micromoles.

#### Determination of reduced glutathione concentration

The content of reduced GSH of the hippocampus tissues, as a non-protein sulfhydryl, was estimated according to the method described^50^. Hippocampus tissues were homogenized in 0.02 M EDTA solution (1 mL/100 mg tissue). Aliquots (400 μl) of tissue homogenate were mixed with 320 μL of distilled water and 80 μl of 50% (w/v) trichloroacetic acid in glass tubes and centrifuged at 3000 rpm for 15 min. Next, 400 μL of each supernatant was mixed with 800 μL of Tris buffer (0.4 M, pH 8.9) and 20 μL of 0.01 M 5,5-dithio-bis (2-nitrobenzoic acid). After shaking the preparation, absorbance was measured at 412 nm by a spectrophotometer. GSH concentration was ascertained via a standard curve of reduced GSH, generated in parallel. The results were expressed as micrograms of GSH per gram of tissue.

#### Determination of malondialdehyde concentration

The levels of MDA in homogenates from each group were measured using the method described^51^, which is based on a thiobarbituric acid reaction. Hippocampus tissues were homogenized with 1.15% cold KCl to prepare 10% homogenates. In brief, 250 μL of each homogenate was added to 1.5 mL of 1% H_3_PO_4_ and 0.5 mL of 0.6% 2-methylpropan-2-ol (aqueous solution). Later, this mixture was stirred and heated in a boiling water bath for 45 min. The preparation was then cooled immediately in an ice water bath, followed by the addition of 2 mL of butan-1-ol. The mixture was shaken and the butan-1-ol layer was separated by centrifugation at 4000 rpm for 15 min. Optical density was determined at 535 and 520 nm, and the difference in optical density between the two estimates was calculated as the 2-methylpropan-2-ol value. MDA concentrations are expressed as nanomoles per gram of tissue.

### ROS production in living microglia and neuronal cultures

#### Microglial cell line

The human microglial cell line, CHME3, was obtained from primary cultures of human embryonic microglial cells by transfection with a plasmid encoding for the large T antigen of SV40. Such cells were previously used in microglial studies demonstrating similar activities related to primary cultures^52^. CHME3 microglia were cultivated as previously described^40^. In brief, cells were cultured in DMEM GlutaMAX^TM^-I, 100 U/mL penicillin, and 100 μg/mL streptomycin supplemented with 10% FBS. Cells were kept at 37 °C, 95% air, and 5% CO_2_ in a humidified incubator. Cells were plated on plastic-bottom culture dishes (μ-Dish 35 mm, iBidi) for live imaging experiments.

#### Hippocampal neuronal cultures

Hippocampal neurons were obtained from E16 C56Bl/6 mice. After dissection in Ca^2+^- and Mg^2+^-free Hank’s Balanced Salt Solution (HBSS, Thermo Fisher Scientific, Waltham, MA), hippocampi were treated with trypsin (0.045% in HBSS) for 10 min at 37 °C (Thermo Fisher Scientific), washed with 10% fetal bovine serum in HBSS to block trypsin activity, and washed further in HBSS to remove serum and prevent the growth of glia. Finally, the hippocampi were transferred to serum-free Neurobasal medium (Thermo Fisher Scientific) supplemented with B27 (1:50, Thermo Fisher Scientific), glutamine (0.5 mM, Sigma-Aldrich, Merck), gentamicin (0.12 mg/mL, Thermo Fisher Scientific) and L-glutamate (25 µM, Sigma-Aldrich, Merck) and mechanically dissociated. Neurons were kept in supplemented Neurobasal medium on coverslips treated with nitric acid and coated with poly-D-lysine (20 µg/cm^2^, P0899, Sigma-Aldrich, Merck) at a density of 8 × 10^4^ cells/cm^2^, at 37 °C in a humidified incubator with 5% CO_2_/95% air for 7–8 days.

#### Transfection using the calcium phosphate co-precipitation method

Hippocampal neurons were transfected on the fifth day *in vitro* (DIV). Towards this end, 2 µg/coverslip of plasmid DNA (mVenus^53^ and HyPer Red ROS Biosensor^54^) were diluted in Tris-EDTA buffer (TE; 10 mM Tris, 1 mM EDTA), pH 7.3, and mixed with a calcium chloride solution in HEPES (2.5 M CaCl_2_ in 10 mM HEPES), pH 7.2. This mixture of DNA/TE/Calcium was added to 2 x HEPES buffered saline (270 mM NaCl, 10 mM KCl, 1.4 mM Na_2_HPO_4_, 11 mM dextrose, and 42 mM HEPES), pH 7.2. Precipitates were allowed to form for 30 min, with vortex mixing every 5 min, to ensure that the precipitates were of similar small sizes. Meanwhile, neurons were incubated with 2 mM kynurenic acid (4-oxo-1H-quinoline-2-carboxylic acid). The precipitate was then added to the neurons, and incubated at 37 °C, 5% CO_2_/95% air, for 3 h. Neurons were then washed with acidic culture medium containing 2 mM kynurenic acid and incubated further for 15 min. Finally, the medium was replaced with the initial culture medium, and the neurons were further incubated in a 37 °C/5% CO_2_ incubator for 48 h to allow protein expression.

Microglia transfection was performed on the second DIV, using the same procedure as for neuronal transfection, using 10 µg of DNA/plate (NFkB pathway inhibitor biosensors^55^ and the HyPer Red ROS Biosensor^54^) with exception of the kynurenic acid incubation for live cell imaging. Assays were performed at least 48 h after transfection to allow protein expression.

#### Live cell imaging and quantification of biosensors

Experiments were performed on a fully-motorized DMI6000B microscope (Leica Microsystems) equipped with filter cubes for monomeric red fluorescent and far-red fluorescent protein mounted into a microscope filter carrousel (Leica fast filter wheels). The excitation light source was a mercury metal halide bulb integrated with an EL6000 light attenuator. Cells were observed with a PlanApo 63 × 1.3NA glycerol immersion objective with a correction ring. An ORCA-Flash 4.0 V2 Digital CMOS camera (Hamamatsu) coupled to the microscope through a 1.3x c-mount adapter was used to acquire images. The LAS X software (Leica Microsystems) controlled all microscope parameters. At each time point, images were sequentially acquired using 2 × 2 camera binning with an exposure time of 800 milliseconds. Time-lapse images were exported as 16-bit tiff files and processed by the FIJI software as described previously^40,56,57^. In brief, background was dynamically subtracted from time-lapses using the rollerball algorithm. Cells were individually segmented and thresholded using PFRET. Whole cell mean grey intensity values for each biosensor was retrieved and plotted.

#### Haemolytic assays

The haemolytic activity of Ocellatin-K1(1–16) and Ocellatin-K1(1–21) peptides was tested using human red blood cells (RBC) as previously described^58^, with some modifications. Briefly, RBCs were collected in EDTA (1.8 mg/mL), washed three times with sterile saline solution (0.85%), and the pellets were resuspended and diluted in the same solution. The RBC suspension was added to an equal volume of each peptide solution at different concentrations (7.8–500 μg/mL). The mixtures were incubated for 30 minutes at 37 °C and then centrifuged at 8000 rpm for 1 min. The supernatants were removed and the value of absorbance (Abs) at 492 nm was measured. Maximum haemolysis was determined by adding 0.1% Triton-X (v/v) to a sample of cells, and saline solution was used as negative haemolysis controls. The haemolysis percentage was calculated as follows: [(Abs peptide − Abs saline)/(Abs Triton − Abs saline)] × 100.

#### Atomic Force Microscopy (AFM) on the human red blood cells

Isolated human erythrocytes (control or treated) were fixed with 2% glutaraldehyde in 100 mM PBS, pH 7.4 for 2 hours while mixing, followed by deposition on poly-L-lysine coated coverslips and eventual washing with ultrapure water. The samples were air-dried overnight before imaging. AFM was performed with a JPK Nanowizard 4 instrument (JPK Instruments AG. Germany), in AC-AFM (tapping) mode, using ACT cantilevers with a resonant frequency of approximately 300 kHz.

#### Statistical analysis

All experimental data was expressed as mean ± standard error of the mean (SEM). Results from saline-treated control animals were used as baseline values. The results obtained from Ocellatin-K1(1–16) and Ocellatin-K1(1–21) or reference drug-treated test groups were compared with those obtained from saline-treated controls. All statistical analyses were performed with GraphPad Prism (version 6.0) software. The statistical significance of the differences between groups of experiments *in vivo* was determined by one-way analysis of variance (ANOVA) and the multiple comparison Student−Newman−Keuls test. The anti-microbial test was analysed using the ANOVA and Sidak test. The differences in the effects of compound treatment compared with control values to cell cultures were analysed by one-way ANOVA with the Bonferroni post-test. ANOVA and *t*-test were used to compare the measurements of haemolytic test. P < 0.05 was considered statistically significant.

## Data Availability

All data generated or analysed during this study are included in this article.
